# Early life factors and dementia risk: A study of adverse childhood experiences and later‐life cognition and behavior

**DOI:** 10.1002/alz.091655

**Published:** 2025-01-09

**Authors:** Dinithi M Mudalige, Dylan X. Guan, Eric E. Smith, Zahinoor Ismail

**Affiliations:** ^1^ University of Calgary, Calgary, AB Canada; ^2^ Hotchkiss Brain Institute, University of Calgary, Calgary, AB Canada; ^3^ Department of Clinical Neurosciences and Hotchkiss Brain Institute, University of Calgary, Calgary, AB Canada

## Abstract

**Background:**

Of the 12 modifiable dementia risk factors established by the Lancet Commission, only one addresses early life. However, the brain is highly plastic in early life. Adverse childhood experiences (ACE)–physical, emotional, and sexual abuse and neglect–can result in long‐term reductions in brain volume. These alterations increase susceptibility to neurodegenerative diseases like Alzheimer’s disease. We investigated associations between the severity of ACE and cognitive and behavioral risk for dementia, operationalized as later‐life cognitive impairment and mild behavioral impairment (MBI), respectively.

**Method:**

Data were from the Canadian Platform for Research Online to Investigate Health, Quality of Life, Cognition, Behavior, Function, and Caregiving in Aging (CAN‐PROTECT) study. Measures included the Childhood Trauma Screener (for ACE), neuropsychological tests (objective cognition), Everyday Cognition (ECog)‐II (subjective cognition), and the MBI Checklist (MBI‐C, later‐life behavioral changes). Linear regressions modeled associations between ACE severity and neuropsychological test scores. Multivariable negative binomial regressions (zero‐inflated, if appropriate) modeled associations between ACE severity and ECog‐II and MBI‐C scores. Models adjusted for age, sex, education, and ethnocultural origin. Clinical diagnosis of depression and/or anxiety were explored as covariates and mediators of the observed associations.

**Result:**

Participants (n = 1102) were 64.6 (SD = 7.3) years old; 883 (80.1%) were female, 290 (26.3%) had a bachelor’s degree or equivalent, and 939 (85.2%) were of European origins. Every 1 standard deviation (SD) increase in ACE score was associated with worse performance on Trail Making B (b = 0.10, 95%CI:0.05–0.16), Switching Stroop (b = ‐0.08, 95%CI:‐0.14—0.03), Paired Associates Learning (b = ‐0.08, 95%CI:‐0.13—0.02), and Digit Span (b = ‐0.08, 95%CI:‐0.14—0.02). Every 1 SD increase in ACE score was associated with higher ECog‐II (b = 1.1, 95%CI:1.0‐1.1, p = 0.007) and MBI‐C (b = 1.2, 95%CI:1.1–1.3, p<0.001) scores, not mediated by previous psychiatric history (ECog II p = 0.16; MBI‐C p = 0.13).

**Conclusion:**

Greater ACE severity predicted more later‐life cognitive and behavioral symptoms, representing higher dementia risk, not mediated by psychiatric history. These findings extend the public health importance of ACE to brain aging and emphasize the importance of incorporating early‐life risk factors into dementia risk assessment. ACE are potential targets for risk reduction and identify persons who should be screened for cognitive and behavioral risk markers.

